# Characterization of Drug Release from Mesoporous SiO_2_-Based Membranes with Variable Pore Structure and Geometry

**DOI:** 10.3390/pharmaceutics14061184

**Published:** 2022-05-31

**Authors:** Frank Baumann, Theresa Paul, Susan Wassersleben, Ralf Regenthal, Dirk Enke, Achim Aigner

**Affiliations:** 1 Rudolf-Boehm-Institute for Pharmacology and Toxicology, Clinical Pharmacology, Faculty of Medicine, Leipzig University, 04107 Leipzig, Germany; frank.baumann2@medizin.uni-leipzig.de (F.B.); ralf.regenthal@medizin.uni-leipzig.de (R.R.); 2 Institute of Chemical Technology, Leipzig University, 04103 Leipzig, Germany; theresa.paul@uni-leipzig.de (T.P.); susan.wassersleben@uni-leipzig.de (S.W.)

**Keywords:** drug release, mesoporous membranes, LC-MS, transdermal drug delivery systems

## Abstract

Transdermal drug delivery systems (TDDSs) play important roles in therapy due to distinct advantages over other forms and types of drug application. While common TDDS patches mainly consist of polymeric matrices so far, inorganic carriers show numerous advantages such as high mechanical stability, possible re-use and re-loading of drugs, and a broad chemical compatibility with therapeutically relevant compounds and chemical enhancers. Mesoporous glasses can be prepared in different monolithic shapes, and offer a particularly wide range of possible pore volumes, pore diameters, and specific surface areas. Further, they show high loading capacities and favorable physical, technical, and biological properties. Here, we explored for the first time monolithic SiO_2_-based carriers as sustained release systems of therapeutic drugs. In an ideally stirred vessel as model system, we systematically analyzed the influence of pore diameter, pore volume, and the dimensions of glass monoliths on the loading and sustained release of different drugs, including anastrozole, xylazine, imiquimod, levetiracetam, and flunixin. Through multilinear regression, we calculated the influence of different parameters on drug loading and diffusion coefficients. The systematic variation of the mesoporous glass properties revealed pore volumes and drug loading concentrations, but not pore diameter or pore surface area as important parameters of drug loading and release kinetics. Other relevant effectors include the occurrence of lateral diffusion within the carrier and drug-specific properties such as adsorption. The structure–property relationships derived from our data will allow further fine-tuning of the systems according to their desired properties as TDDS, thus guiding towards optimal systems for their use in transdermal drug applications.

## 1. Introduction

Transdermal application systems (transdermal drug delivery systems, TDDSs) play important roles in drug therapy, with marked increases in their approval and medical use over the past decades [1]. Major advantages over other forms and types of application (oral, topical, injection) include achieving more constant levels of active ingredient over longer time periods through sustained/controlled release. By avoiding concentration peaks, this leads to improved drug tolerability and efficacy due to prolonged maintenance of therapeutic drug levels in the blood [2]. Furthermore, the gastrointestinal tract is not the site of drug entry, and thus it is less exposed as compared with oral application. Other advantages include lesser first pass effects, more uniform plasma levels, lower dosing frequencies, minimally invasive application, and improved compliance.

Common TDDSs as patches, i.e., engineered, external continuous and long-acting dosage forms [3], mainly rely on various polymers so far, including natural polymers, synthetic elastomers, and synthetic polymers [1]. They can be distinguished between first-generation and second-generation TDDSs, which include skin permeability enhancers, or a third-generation TDDS employing novel chemical enhancers, electroporation, ultrasound, microneedles, thermal ablation, or microdermabrasion [4,5,6]. Examples include TDDSs based on cellulose, chitosan, gelatin, xanthan gum, or various synthetic polymers like poly(lactic-co-glycolic acid (PLGA), poly(N-isopropyl-acrylamide), polyacrylates, and many others [5,7,8]. Since 1979, several transdermal products have received market approval [9,10].

Issues related to currently used TDDSs include limited loading capacities esp. in the case of drugs requiring higher plasma levels, limited efficacy when using hydrophilic drugs, as well as possible chemical interactions/compatibility of the drug or a chemical enhancer with the TDDS. Other requirements include high mechanical and storage stability, complete drug release, and perhaps a biphasic release profile and possible re-use upon re-loading of the TDDS [11]. The full potential of TDDS will thus rely on the development of novel systems, which are usable for a broad spectrum of different drugs, solvents, and chemical enhancers, and offer favorable physical, technical, and biological properties.

In the last decade, mesoporous silica nanoparticles (MSNs) gained enormous scientific interest in the field of controlled drug release. MSNs allow for relatively high loading concentrations of drug molecules while displaying the physicochemical stability known for silica. Furthermore, amorphous silica materials exhibit high biocompatibility, are non-toxic, and are widely used as adjuvant in pharmaceutical technology [12,13,14,15]. Thereby, MSNs obtain significant advantages over other drug carrier systems like micelles, liposomes, and polymers, which suffer from poor thermal and chemical stability [12,16]. While mesoporous silica has been extensively investigated as a carrier for oral drug delivery, this work aimed to combine the advantages of an inorganic silica material with those of transdermal drug delivery routes. Thereby, porous glasses were studied as monolithic TDDS carriers for the first time.

Porous glasses have been used in various application fields such as in catalysis, ion exchange, adsorption, and sensor technology [17,18,19]. Their chemical durability and surface, mainly determined by silanol groups that can be functionalized by a wide variety of organosilanes as coupling agents, are particularly interesting for potential applications in drug delivery [20]. Furthermore, porous glasses are characterized by narrow pore size distributions, with tunable pore diameters in the range of 1 to 1000 nm for specific applications as well as adjustable pore volumes. These glasses can be prepared in various monolithic shapes like beads, flat membranes, fibers, and tubes [17,18,19,20]. As a result, porous glasses are suitable as model systems for fundamental studies on the diffusion of drugs in porous materials and their sustained release kinetics.

To the best of our knowledge, no studies exist in the scientific literature on a sustained release of therapeutic substances from monolithic SiO_2_-based carriers, which can be achieved over a long period of time. In particular, this also applies to systematic investigations of the influence of pore diameter, pore volume, tortuosity of the pore system, and the dimensions of glass monoliths on sustained release. Such systems have not yet been investigated for transdermal applications (e.g., as a special patch) in terms of continuous long-term release. This work comprehensively analyzed the release behavior of drugs from a larger set of flat porous glass membranes, with systematic variation of their properties.

## 2. Materials and Methods

### 2.1. Materials

Solvents (acetonitrile (Baker; Gliwice, Poland), methanol (Promochem; Wesel, Germany), DMSO (dimethyl sulfoxide; Roth, Karlsruhe, Germany), and bi-distilled water (Honeywell; Seelze, Germany)) were of LC-MS grade. Anastrozole, xylazine (HCl), levetiracetam, and imiquimod were obtained from TCI (Eschborn, Germany), d_12_ anastrozole and d_9_ imiquimod were from Cayman Chemical (Ann Arbor, MI, USA), d_6_ xylazine, flunixin and d_3_ flunixin were obtained from Sigma (Taufkirchen, Germany), and d_3_ levetiracetam was purchased from Toronto Research Chemicals (North York, ON, Canada). Table 1 gives an overview of the drugs used in this study and important drug properties.

For LC-MS, a SpectraSystem apparatus (Finnigan/Thermo Fisher, Waltham, MA, USA) was employed, with pump (SpectraSystem P4060), degasser (SpectraSystem SCM 1000), interface (SpectraSystem SN 4000), and SSQ7000 single-quadropole mass spectrometer. The software was XCalibur Version 1.3 and SSQ Tune Version 1.1 (San José, CA, USA).

### 2.2. Preparation of Mesoporous Membranes

Three different starting glasses with compositions (70-X)SiO_2_·(23 + X)B_2_O_3_·7Na_2_O (wt.%) with X = 0–7.5 wt.% were prepared by stoichiometric mixing of analytical grade SiO_2_ (Alfa Aesar 99.5%), B_2_O_3_ (Alfa Aesar 98%), Na_2_CO_3_ (Roth 99.5%), and NaNO_3_ (Merck 99.99%), prior to melting at 1400 °C for 2 h in an electric furnace (LHT 04/17, Nabertherm, Lilienthal, Germany). To enhance the chemical homogeneity, the glasses were crushed, ground, and re-melted at 1400 °C for 2 h. The glasses were poured on a preheated brass-casting mold. To avoid the formation of tensions in the material, the solidified glass blocks were annealed and subsequently cut into 1 × 1 × 5 cm geometry. Selected glass blocks were thermally treated at 535 °C for 24 h to induce phase separation, thereby generating an interconnected pore network. The resulting glass blocks were cut into 1 × 1 cm membranes (plates) with a thickness of 200, 300, or 500 µm, respectively, using a precision saw (Buehler IsoMet High Speed Pro, ITW Test & Measurement, Leinfelden-Echterdingen, Germany) and subsequently leached for 2 h in 1 M/3 M HCl at 90 °C for removing the sodium-rich borate phase. Selected membranes were further leached with a 0.5 M NaOH solution for 2 h at room temperature, for removing colloidal silica deposits (secondary silica) in the pore structure. After each leaching step, the membranes were washed with deionized water until pH = 7 and subsequently dried.

### 2.3. Characterization of Mesoporous Membranes

The textural properties of porous membranes were analyzed by nitrogen sorption and mercury intrusion porosimetry. For nitrogen sorption measurements, 30 mg of sample mass were activated at 250 °C under ultrahigh vacuum for 10 h. The experiments were performed using a Quantachrome autosorb iQ (Quantachrome Instruments, Anton Paar QuantaTec, Boynton Beach, FL, USA) at a temperature of −196 °C in the relative pressure range 0–0.995 p/p_0_. The pore width distribution was determined by density function theory (DFT) [21]. The specific surface area was calculated based on the Brunauer–Emmett–Teller (BET) method in the pressure range from 0.1 to 0.4 p/p_0_ [22]. Total pore volume was determined from the adsorption branch of the isotherm at a relative pressure of 0.995 p/p_0_.

In addition, selected samples were analyzed by mercury intrusion porosimetry. The samples were degassed (0.2 mbar at RT) with a PASCAL 140 porosimeter. Mercury intrusion was performed with a PASCAL 440 porosimeter (ThermoScientific/POROTEC, Hofheim, Germany) with a pressure range of 1 to 1000 bar. Mercury surface tension was assumed at 0.484 N/m, and its contact angle was set to 141.3°. The pore width distribution was determined by applying the Washburn equation, assuming a cylindrical pore model.

### 2.4. Membrane Loading and Drug Release

Prior to drug loading, residual moisture in the mesoporous membranes was evaporated (100 °C for 30 min). Drugs were dissolved in acetonitrile (anastrozole), methanol (xylazine and levetiracetam), DMSO (flunixin), and 90% acetonitrile/1% *v*/*v* formic acid (imiquimod), prior to incubating membranes in 0.5 mL of this solution for 6 h at room temperature in 24-well plates. After loading, membranes were gently wiped to remove excess solution on the outer surface.

Prior to drug release, the loaded membranes were dried by evaporating residual solvent at 100 °C for 15 min (acetonitrile, methanol) or 120 °C for 30 min (DMSO). The release tests were carried out in a stirred 50 mL tube containing 20 mL bi-distilled water. Bi-distilled water was preferred over physiological solutions like phosphate-buffered saline (PBS) since from the material (intertness) and drug side it does not make a difference, and PBS would have required an additional liquid–liquid extraction process prior to LC-MS, potentially reducing accuracy of the determination of drug concentrations. 200 µL samples were taken at 0, 1, 2, 3, 4, 5, 10, 15, 30, 45, 60, 120, 180, and 300 min and replaced by 200 µL bi-distilled water to keep the total volume constant over time. To determine the total amount of drug loading in the pore structure (100% value), mesoporous membranes were further incubated for at least 24 h, with subsequent sample taking. To ensure complete release after this time period, membranes were then transferred to acetonitrile (anastrozole, flunixin, and imiquimod) or methanol (xylazine, levetiracetam) and incubated for an additional 2 h. From this, the residual drug concentrations were measured, which were negligible for all drugs except for xylazine (~2–7%). Thus, drug concentrations after 24 h were set as the total drug loading concentration (100%), with the exception of xylazine.

### 2.5. Determination of Drug Concentrations

Drug concentrations were determined using liquid chromatography-mass spectrometry (LC-MS). An Atlantis column (2.1 mm × 150 mm + precolumn 5 mm; Waters, Eschborn, Germany) was used, with the flow rate set to 0.2 mL/min. Two different solvents were used: 40% acetonitrile + 0.1% formic acid (levitiracetam, xylazine, imiquimod) and 55% acetonitrile + 0.1% formic acid (anastrozole, flunixin). The single ion mode in positive modus was used for all measurements. Deuterated substances were applied as internal standards: d_12_-anastrozole, d_9_-imiquimod, d_6_-xylazine, d_3_-levitaracetam and d_3_-flunixin.

Parameters for all substances were as follows: 40 psi nitrogen 5.0 as sheath gas and 10 psi nitrogen as auxiliary gas, 220 °C capillary temperature, 4.5 kV in positive modus ionization voltage, and 1400 V multiplier voltage. The current ionization-dissociation voltage (CID) was 5 V for levetiracetam, 10 V for xylazine and imiquimod, and 20 V for flunixin and anastrozole, respectively. The drugs were measured at the following mass units (Da): levetiracetam 171 (internal standard: 174), xylazine (221; 227), imiquimod (241; 250), anastrozole (225; 237), and flunixin (297; 300). All molar masses refer to MH^+^ except for anastrozole where fragment ions were used due to a better intensity-to-noise ratio. Representative chromatograms and calibration curves are shown in the Supplementary Material Figure S1.

### 2.6. Calculation of Diffusion Coefficients

The diffusion coefficient was calculated from the slope of drug release within the first three minutes. The following equation [23] was used:

(1) $$D=\frac{J\;\cdot \;d/2}{c_{0}}$$

where *J*, substance flow from the membrane in µg/(min cm^2^); *d*, membrane thickness; *c*_0_, initial concentration in the membrane (drug amount/pore volume). In case of mesoporous membranes with one side sealed, *d*/2 was replaced by *d* in Equation (1). The parameter *J* was determined from the slope (µg/min × mL) × acceptor fluid volume (mL)/area (cm^2^).

## 3. Results

### 3.1. Properties and Characterization of Mesoporous Membranes

**Figure 1:**
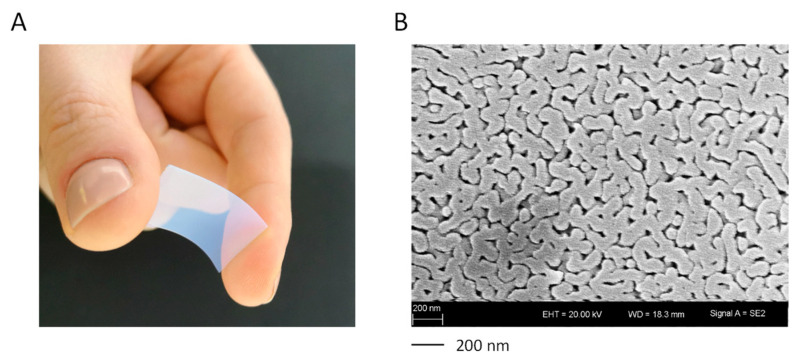
(**A**) Macroscopic shape and (**B**) SEM picture (right) of a flexible porous membrane (CPG-300-0.40).

Porous glasses are amorphous materials with SiO_2_ contents of >96 wt.% and represent the products of leached phase separated alkali borosilicate glasses. These were first obtained in the 1930s as an intermediate product of the VYCOR process [24]. All glass compositions used in this work lie in the immiscibility region of the ternary system of B_2_O_3_–SiO_2_–Na_2_O. Due to thermal treatment, the glasses undergo diffusion processes leading to a spinodal phase separation into a silica phase and a sodium-rich borate phase, which form an interconnecting and interpenetrating network. The temperature and length of the thermal treatment as well as the composition determine the extent of phase separation, and thereby the resulting pore diameter and pore volume. Subsequently, the sodium-rich borate phase formed by phase separation can be leached by treatment with water or mineral acids. A porous sponge-like network of silica is obtained (Figure 1) [17,18,19].

The denominations and textural properties of the mesoporous glass membranes used in this study are shown in Table 2, with the denomination of “CPG-xxx-y.yy” indicating “CPG = controlled porous glass”, “xxx = the membrane thickness (µm)”, and “y.yy = the pore volume (cm^3^/g)”.

Pore diameters and pore volumes were derived from nitrogen sorption measurements for all membranes with pore sizes below 30 nm. Otherwise, the textural properties were obtained using mercury intrusion porosimetry. Nitrogen sorption isotherms showed type IVa isotherms with an H1 hysteresis, indicating mesoporous materials with a narrow pore size distribution (Supplementary Material Figure S2A). The steep, narrow hysteresis loop suggested “ink-bottle” pores, where the pore mouth and the pore cavity are similar in width [25]. The pore structure was completely filled with nitrogen, as indicated by the saturation plateau at high p/p_0_ values, thereby further indicating pure mesoporous materials. Correspondingly, through the DFT method narrow pore width distributions were obtained. In the example of the glass membrane CPG-300-0.87, a peak value of 17 nm in the mesoporous range was determined (Supplementary Material Figure S2B; see Table 2 for all values). Similar nitrogen sorption isotherms were obtained for the other porous glass membranes (data not shown). Mercury intrusion porosimetry further indicated a narrow pore size distribution in the mesopore range (Supplementary Material Figure S2C).

Post-treatment with NaOH after acidic leaching (membranes with pore sizes > 10 nm) showed a significant increase in pore volume and, due to the larger pore diameters, a decrease in BET surface areas. By varying compositions and post-treatments, porous membranes were prepared with similar pore diameters but increasing pore volumes. This allowed the generation of an ideal model system for systematic investigations of the relationship between structural properties and drug release kinetics.

### 3.2. Characterization of Drug Loading Properties: Effects of Pore Volume, Pore Surface and Loading Concentration

**Figure 2:**
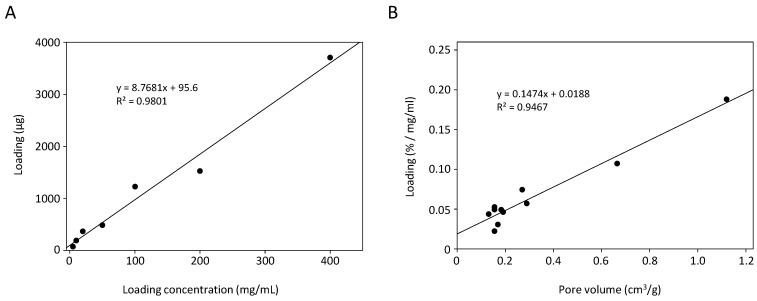
Drug loading of anastrozole as function of (**A**) loading concentration, exemplified for glass membrane CPG-200-0.16, and (**B**) pore volume of different membranes.

Drug loading properties of the mesoporous membranes, with CPG-200-0.16 as representative example, were first analyzed using anastrozole as test substance. Drug loading was found to be linearly dependent on the loading concentration, i.e., the drug concentration in the incubation solution. This was true over a wide concentration range of 1–400 mg/mL (Figure 2A).

A linear correlation was also found between drug loading and pore volume. For this, mesoporous membranes with 300 µm thickness and specific surface areas in the range between 27–240 m^2^/g were used, whereby drug loading was plotted against pore volume (Figure 2B). The coefficient of determination was R^2^ = 0.9467. In contrast, linear regression analysis revealed that variation of the specific surface area showed no correlation with drug loading. This also indicates, in the case of anastrozole, the absence of (pore) surface effects on overall loading, like drug adhesion to the pore walls. The following equation was derived from SigmaPlot 14.0, performing a multilinear regression with the following settings: *Drug loading*, dependent variable, and parameters *pV* and *A_BET_*, independent variables:

(2) $$Drug\;loading=1.72\cdot 10^{-2}+\left(1.46\cdot 10^{-1}\cdot pV\right)+\left(1.69\cdot 10^{-5}\cdot A_{BET}\right)$$

with *pV*, pore volume (cm^3^/g) and *A_BET_*, pore surface area (m^2^/g). It indicates the pore volume as the critical parameter for loading capacities of the mesoporous membranes (Supplementary Material Figure S3).

### 3.3. Drug Release Properties as Function of Various Mesoporous Membrane Properties

**Figure 3:**
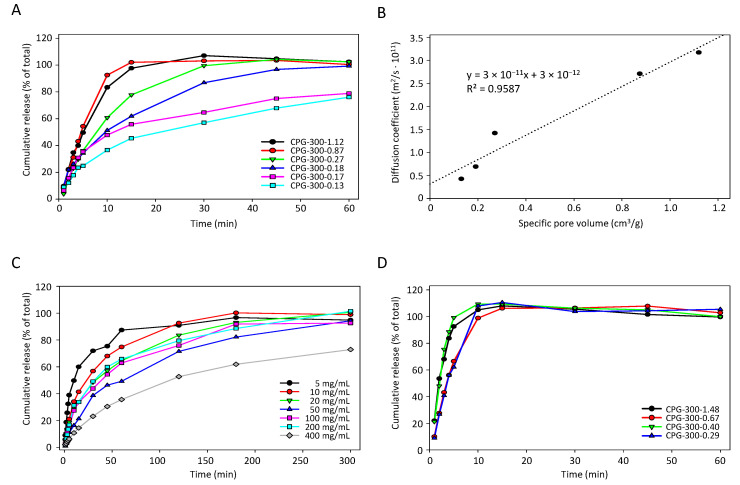
Release properties of anastrozole from mesoporous glass membranes (loading concentration: 10 mg/mL). In the case of membranes with small pore diameters between 4–17 nm, anastrozole release is dependent on (**A**) pore volumes, which (**B**) linearly affect the diffusion coefficient, and (**C**) loading concentration. (**D**) The dependence of drug release on pore volume is not seen in the case of membranes with large pore diameters.

Next, we determined cumulative drug release properties of the mesoporous membranes and their dependence on pore architecture. Again, anastrozole was used as the model substance. Drug concentrations were determined by LC-MS, with a lower quantitation limit of 0.1 µg/mL and a measurable linear concentration range of 0.1–100 µg/mL (Supplementary Material Figure S1). The cumulative drug release curves revealed an initially time-dependent increase over max. 120 min, before reaching the plateau of 100% drug release. Notably, in the case of small pore diameters the release kinetics were dependent on the pore volume (Figure 3A, Supplementary Material Figure S4A).

More specifically, when selecting mesoporous membranes with comparable pore diameters between 4–17 nm, 100% drug release from pores with large volumes (0.17 or 0.27 cm^3^/g) was observed within ~60 min while in the case of small pore volumes (e.g., 0.13 cm^3^/g), the plateau was only reached after >150 min (Supplementary Material Figure S4A). The higher magnification of the initial time range (0–60 min) and use of more mesoporous membranes with similar pore diameters but an even broader range of pore volumes (0.13–1.12 cm^3^/g) revealed the direct dependence of cumulative drug release on pore volume in the case of all mesoporous membranes (Figure 3A). In fact, the determination of the diffusion coefficient indicated a linear correlation with the pore volume (Figure 3B; R^2^ = 0.9587). In contrast, no clear association between drug release and pore diameter was detected (Supplementary Material Figure S4B).

The cumulative release was also found to be dependent on the loading concentration, as shown in the case of the glass plate CPG-200-0.16. While in the case of low mesoporous membrane loading, as indicated by loading concentrations of 5.0 or 10.0 mg/mL, the 100% plateau was essentially reached after <180 min, the highest 400 mg/mL loading achieved an only ~60% drug release at this time point, with little progress thereafter (Figure 3C). This indicates the inhibition of drug diffusion and release from the pores at high concentrations, and identifies pore volumes as another critical parameter of drug release from small pores. Quite in contrast, when switching to mesoporous membranes with large pore diameters (37–48 nm), this dependence of cumulative drug release on pore volume was not seen. Here, the variation of the pore volumes between 0.4–1.484 cm^3^/g led to only negligible differences with no correlation (Figure 3D). It can thus be concluded that, except for very porous membranes with large pore diameters, sustained drug release is directly dependent on pore volumes and drug loading.

The major impact of the pore volume on the diffusion coefficient in smaller pores and comparatively few effects of diameter variations under these conditions were also confirmed by multilinear regression analysis. The dependence of the diffusion coefficient on membrane thickness, pore volume, pore diameter, and loading concentration was determined as described by Equation (3), which was derived from SigmaPlot 14.0, performing a multilinear regression with the following settings: *D*, dependent variable, and parameters *d*, *pV*, *pD* and $c_{0}$, independent variables:

(3) $$D=1.616\cdot 10^{-11}+\left(6.04\cdot 10^{-11}\cdot d\right)+\left(3.153\cdot 10^{-11}\cdot pV\right)+\left(3.636\cdot 10^{-13}\cdot pD\right)-\left(8.056\cdot 10^{-15}\cdot c_{0}\right)$$

where *D*, diffusion coefficient (m^2^/s), *d*, membrane thickness (mm), *pV*, pore volume (cm^3^/g), *pD*, pore diameter (nm) and $c_{0}$, loading concentration (mg/mL).

For example, when selecting a membrane thickness of 0.3 mm and a 10 mg/mL anastrozole loading concentration, this led to the diffusion coefficient being dependent on the pore volume and pore diameter as follows:

(4) $$D=-6.659\cdot 10^{-13}+\left(1.739\cdot 10^{-11}\cdot pV\right)+\left(8.112\cdot 10^{-13}\cdot pD\right)$$

where *D*, diffusion coefficient (m^2^/s), *pV*, pore volume (cm^3^/g) and *pD*, pore diameter (nm).

**Figure 4:**
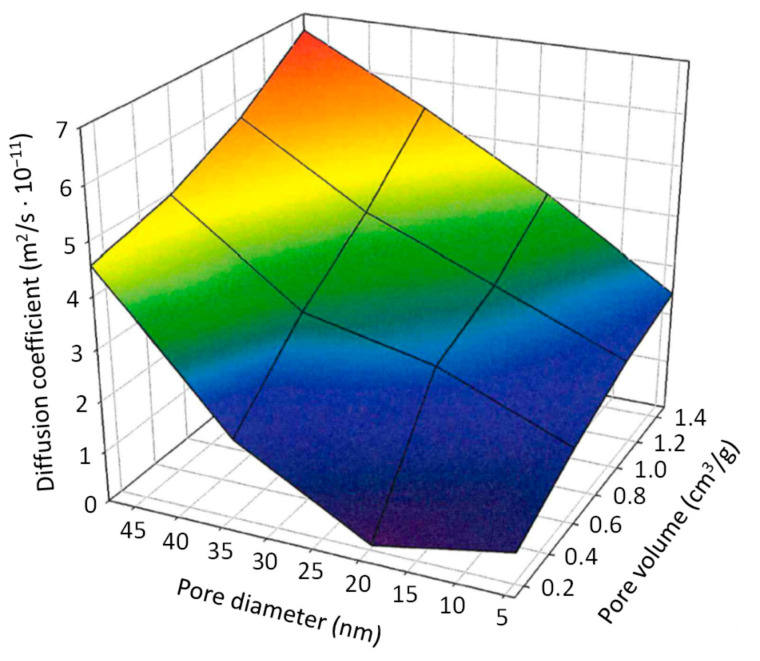
Three-dimensional plot for depicting alterations in the diffusion coefficient vs. pore volume and pore diameter. Details: see text.

The major impact of the pore volume (10^−11^) on the diffusion coefficient in smaller pores and comparatively few effects of diameter variations (10^−13^) under these conditions are shown in Figure 4.

### 3.4. Lateral Diffusion within Mesoporous Membranes

**Figure 5:**
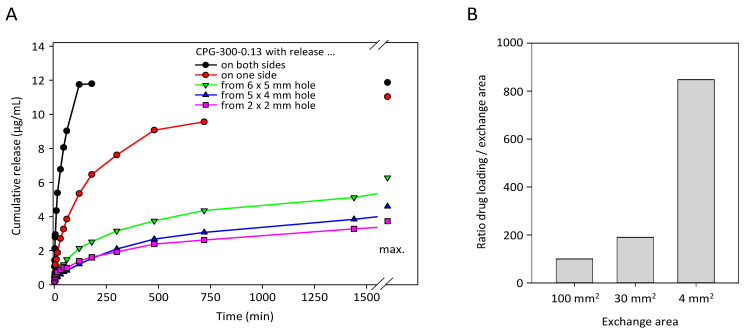
Anastrozole release from the sample CPG-300-0.13 with small pore diameter (9 nm) and small pore volume (0.13 cm3/g). (**A**) Drug release is substantially slowed down when sealing one side (red vs. black) and even more when partially sealing the second side, leaving small holes at sizes indicated in the figure. (**B**) The calculation “drug loading/exchange area”, however, reveals drug amounts well above values to be expected from the small available exchange areas, indicating the contribution of the sealed parts of the mesoporous membranes to drug loading and drug release and thus demonstrating lateral drug diffusion.

To further define drug loading and release properties of mesoporous membranes in dependence of surface area available for drug release, membrane CPG-300-0.13 was selected and defined areas were sealed with epoxy resin, so that release area, but not the pore volume, was restricted. The above results from unsealed membranes were then compared with the corresponding mesoporous membranes after sealing one side, or sealing both sides with an only small hole on one side left unsealed. As to be expected, the still comparatively rapid drug release from a mesoporous membrane with small pore diameter (9 nm) and small pore volume (0.13 cm^3^/g) was substantially slowed down when sealing one side (Figure 5A). This was even more so when the second side was sealed as well, only leaving a small hole of defined size (5 × 6, 4 × 5 or 2 × 2 mm) open for drug release.

Interestingly, however, when analyzing the partially sealed glass plates already for their drug loading capacity, drug amounts in these mesoporous membranes were found to be well above values to be expected from the small available exchange areas. This was particularly obvious when calculating the ratios “drug loading/exchange area”, which substantially increased with smaller areas (Figure 5B). This demonstrates that the sealed parts of the membranes contributed to drug loading as well, by a lateral diffusion of substance from the unsealed area of free exchange into the sections of the glass plate underneath the sealing. Since this lateral diffusion is a slower process compared with the direct vertical drug release, this also contributed to the profound decrease in drug release kinetics observed in Figure 5A.

### 3.5. Dependence of Loading and Release Characteristics on Drug Properties

While the analyses so far were focused on anastrozole, an aromatase inhibitor for treatment of hormone-dependent breast cancer in postmenopausal women, studies were now extended towards other drugs for other indications (Table 1). Drug candidates were selected which could benefit from a sustained release system in their medical use and which cover a broader range of different molecular properties, in particular hydrophilicity/hydrophobicity and presence of protonable groups/heteroatoms (Table 1). 

When comparing different drugs in the same membrane (CPG-300-0.13), major and in fact almost 20-fold differences in drug loading were found. More specifically, imiquimod showed the highest loading capacity, followed by anastrozole, levetiracetam, xylazine and flunixin (Table 3, left). In contrast, when switching to membranes with high porosity (CPG-300-0.40), these differences were largely lost (Table 3, right).

This demonstrates that in larger pores the loading is essentially independent of the nature of the drug, while very small pores allow more profound drug interactions with the pore wall. Thus, the particularly high loading capacity of imiquimod in the small pores (well above the other drugs and twice as high as its loading in the larger pores) may result from its partial protonation at the aromatic amine, leading to electrostatic interactions with the negatively charged membrane surface. In contrast, the carboxylic group of flunixin may lead to partial negative charges and thus electrostatic repulsion from the pore wall, leading to particularly low loading capacity in the small pores, well below the value in the larger pores.

**Figure 6:**
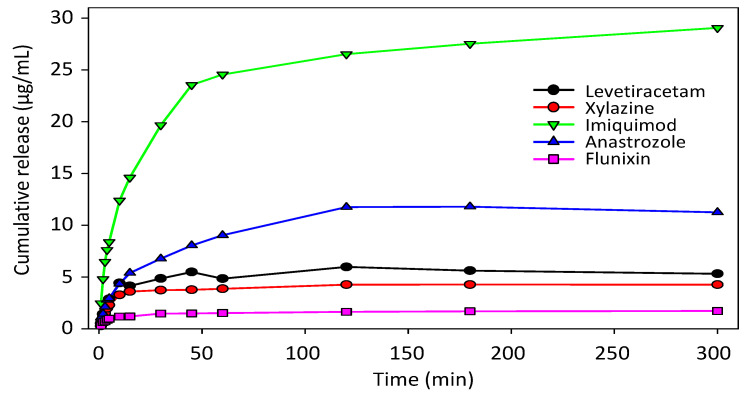
Release properties of various different drugs from the sample CPG-300-0.13.

Differences between the individual drugs were also seen with regard to release kinetics (Figure 6, Table 4).

Based on the differences in their loading, maximum release values in membranes with small pore diameter (CPG-300-0.13) differed to a great extent, with low values in the case of flunixin, xylazine, and levetiracetam. These drugs, however, also reached their plateau at very early time points (<30 min), as compared with anastrozole and especially imiquimod where 100% values were higher, but plateau levels were only reached after ~120 min or >300 min, respectively. These differences were also reflected by the diffusion coefficients, which were 2–4-fold lower in the case of imiquimod and anastrozole as compared with flunixin, xylazine and levetiracetam (Table 4).

**Figure 7:**
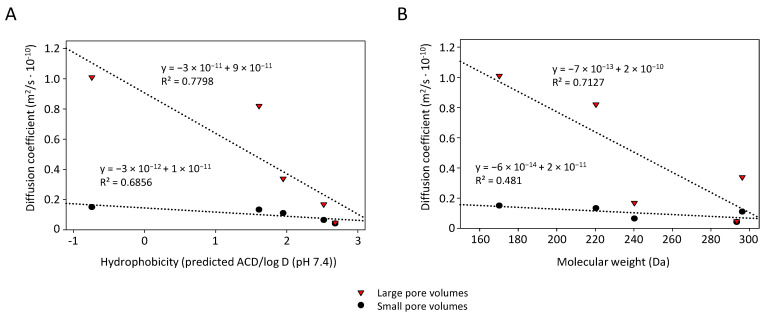
Correlation between diffusion coefficient and (**A**) drug hydrophobicity and (**B**) molecular weight for glass membranes CPG-300-0.13 (small pores; blue) and CPG-300-0.40 (large pores; red).

In the case of the larger pores, diffusion coefficients were overall higher and differences between the individual drugs did not precisely follow the same pattern. Hence, other drug properties may contribute to differences in drug release as well. This was also confirmed when plotting the diffusion coefficient against the hydrophobicity (Figure 7A). While in the case of both small and large pores some correlation was found, R^2^ values were rather low. Likewise, some dependence of the diffusion coefficient on the molecular weight of the drug was observed (Figure 7B). None of these parameters can explain the fact that in the case of xylazine even the long > 24 h membrane incubation did not lead to 100% drug release, with residual 2–7% only recovered after an additional methanol extraction. In the case of the other drugs, similar post-treatment was not required for full recovery. This indicates stronger interactions of xylazine with the pore wall influencing its release from the pores as well.

## 4. Discussion

For therapeutic use, mesoporous silica carriers can offer several advantages over existing TDDSs, including a particularly wide range of possible pore volumes, tortuosities and pore surfaces, high mechanical and storage stability, high loading capacity, possible re-use for re-loading, broad chemical compatibility with various therapeutically relevant compounds and chemical enhancers, as well as favorable physical, technical, and biological properties. Several of these positive properties have already been explored in the case of mesoporous nanoparticles, including their chemical and physical stability, broad and reproducible variation of important parameters related to pore architecture, and their excellent biocompatibility. Notably, these also apply to the macroscopic preparations, and we have now extended the use of mesoporous silica systems towards macroscopic sustained release systems. However, these are based on substantially different geometric forms, for addressing other forms of delivery and transfer, for providing other release profiles, and for being capable of delivering larger drug amounts. Thus, the membranes analyzed here go well beyond what has been described before.

The fundamental influence of pore volumes and pore architecture, including the occurrence of lateral diffusion within the carrier as another important parameter of loading and release kinetics, is highlighted in this study and allows for further fine-tuning of the systems. In fact, structure–property relationships as derived from our data can guide towards optimal systems for use in transdermal drug applications. Preliminary data also confirm high biocompatibility. In vitro experiments indicate excellent attachment and growth of different cell lines (with adjacently growing tumor cell lines being used as models) on the glass membranes, with attachment and growth rates at least similar to the surrounding plastic surface (data not shown). Notably, this was found to be independent of the exact parameters of the glass membranes (pore architecture etc.) and applied to all membranes tested. Thus, the choice of the defined glass preparation can exclusively rely on the desired release profile, without having to consider possible (adverse) biological effects.

However, the release environment present on the skin differs from the submerse conditions employed in this study [1,6]. Release profiles from the TDDS will be slower when used as skin patches, possibly favoring the systems which have shown rather fast release properties in this study. On the other hand, very slow and long-term release characteristics may be therapeutically desired for certain drugs. In this context, the chemical functionalization of mesoporous membranes could be of particular interest, for exploring drug adsorption–desorption as an additional effector of drug loading and release. This is particularly feasible since mesoporous silicas readily allow for a wide range of chemical derivatization without impairing their overall stability, integrity, or other fundamental properties. Indeed, our studies already indicate the possibility of (drug-dependent) interactions with subsequent alterations in drug loading and release. 

Beyond thin membranes, mesoporous silica systems can also be used for generating inorganic textiles or other macroscopic structures. This may be of particular interest when considering issues of (uneven) skin surface and the requirement of realizing a close interaction of the patch with the skin as direct, homogenous, and reproducible as possible. Again, however, pores in these systems will strongly resemble their counterparts in the model membranes used here; thus, our findings presented in this study will also provide the basis for translation into these more complex systems.

The use of a rather wide variety of drugs with different physicochemical properties in this paper, esp. in regards to hydrophilicity/lipophilicity and presence/absence of protonatable heteroatoms for interaction with the membrane surface, confirms the expected broad applicability of our systems for (transdermal) drug delivery. It is tempting to speculate that more complex pore architectures will also allow for more sophisticated, e.g., biphasic, drug release profiles. The use of physical stimuli may further extend the possibilities of fine-tuning and controlling drug release, as already seen in other, for example, thermoresponsive, systems. Notably, these stimuli may not only act onto the skin for inducing alterations that lead to enhanced drug uptake, but may also affect the patch itself. While this is also true for some already available TDDSs, it should be noted that the systems presented here rely on inorganic material. Therefore, other stimuli are readily feasible as well, such as the modification/spiking of the carriers with electrically active components, to generate electro-responsive systems for digitally controlled drug release. Again, these modifications will rely on the previous definition of structure–release properties of unmodified pores, which have been explored here and are presented in this study.

## Figures and Tables

**Table 1:** Drugs used in this study and important drug properties.

Drug	Therapeutic Use	Hydro-/Lipophilicity (log P)	Mol. Mass (g/mol)	Required Drug Levels
Anastrozole	Treatment of hormone-dependent breast cancer in postmenopausal women	lipophilic (2.68)	293.37	systemic; low
Xylazin	Veterinary medicine: sedative/analgesic/ muscle relaxant	intermediate (1.61)	220.33	systemic; low
Imiquimod	Immune modulator; superficial basal cell carcinoma, actinic keratosis, cutaneous warts	lipophilic (2.52)	240.30	local
Flunixin	Veterinary medicine: non-opioid analgesic	intermediate (1.95)	296.25	systemic; high
Levetiracetam	Antiepileptic (inhibitor of glutamate release)	hydrophilic (−0.74)	170.21	systemic; high

**Table 2:** Designations and textural properties of the mesoporous membranes used in this study.

Designation	Pore Diameter (nm)	Pore Volume (cm^3^/g)	BET Surface Area (m^2^/g)	Thickness (µm)
CPG-300-0.13	9	0.13	137	300
CPG-300-0.17	4	0.17	191	300
CPG-300-0.18	4	0.18	154	300
CPG-300-0.27	11	0.27	149	300
CPG-300-0.29	41	0.29	27	300
CPG-300-0.40	48	0.40	27	300
CPG-300-0.67	37	0.67	113	300
CPG-300-0.87	17	0.87	159	300
CPG-300-1.12	17	1.12	240	300
CPG-300-1.48	44	1.48	150	300
CPG-200-0.16	7	0.16	121	200
CPG-500-0.30	17	0.30	138	500
CPG-500-0.19	28	0.19	66	500
CPG-500-0.62	100	0.62	18	500

**Table 3:** Loading capacities (in %/mg/mL) for various drugs at two pore architectures.

Pore Architecture	0.131 cm^3^/g and 9 nm (CPG-300-0.13)	0.4 cm^3^/g and 48 nm (CPG-300-0.40)
Drug		
Anastrozole	0.0436	0.0510
Flunixin	0.0066	0.0392
Levetiracetam	0.0207	0.0374
Imiquimod	0.1117	0.0524
Xylazine	0.0172	0.0444

**Table 4:** Diffusion coefficients for various drugs at two pore architectures.

Pore Architecture	0.131 cm^3^/g and 9 nm (CPG-300-0.13)	0.4 cm^3^/g and 48 nm (CPG-300-0.40)
Drug		
Anastrozole	4.25 × 10^−12^ m^2^/s	4.82 × 10^−11^ m^2^/s
Flunixin	1.11 × 10^−11^ m^2^/s	3.39 × 10^−11^ m^2^/s
Levetiracetam	1.51 × 10^−11^ m^2^/s	1.01 × 10^−10^ m^2^/s
Imiquimod	6.57 × 10^−12^ m^2^/s	1.69 × 10^−11^ m^2^/s
Xylazine	1.34 × 10^−11^ m^2^/s	8.21 × 10^−11^ m^2^/s

## Data Availability

Not applicable.

## Supplementary Materials

The following supporting information can be downloaded at: https://www.mdpi.com/article/10.3390/pharmaceutics14061184/s1, Figure S1: Determination of drug concentrations by liquid chromatography-mass spectrometry (LC-MS). Representative chromatograms of (A) blank value and internal standard, and (B) anastrozole, with the limit of quantitation at 0.1 µg/mL. (C) Calibration curve for anastrozole, in the range of 0.1–100 µg/mL; Figure S2: (A) Selected nitrogen adsorption-desorption isotherms and (B) pore size distribution by DFT method (right) of membranes (here: CPG-300-0.87). (C) Selected pore size distribution obtained by mercury intrusion porosimetry of membranes (here: CPG-300-0.40); Figure S3: Three-dimensional plot for depicting the dependence of drug loading on pore volume and pore diameter. Figure S4: Anastrozole release from sample CPG-300-0.13 with small pore diameter (9 nm) and small pore volume (0.13 cm^3^/g). (A) Cumulative drug release over a long time period of 300 min. (B) No association is observed between drug release and pore diameter.
