# Supplementary material for: Characterization of Drug Release from Mesoporous SiO2-Based Membranes with Variable Pore Structure and Geometry
Source: Pharmaceutics. 2022 May 31;14(6):1184. doi: 10.3390/pharmaceutics14061184 (PMC9230061; doi:10.3390/pharmaceutics14061184)
Supplement: Supplementary file 1 [file pharmaceutics-14-01184-s001.zip › pharmaceutics-1740727-supplementary.pdf]

Supplementary Materials: Characterization of Drug Release from Mesoporous SiO2-Based Membranes with Variable Pore Structure and Geometry

Frank Baumann, Theresa Paul, Susan Wassersleben, Ralf Regenthal, Dirk Enke and Achim Aigner

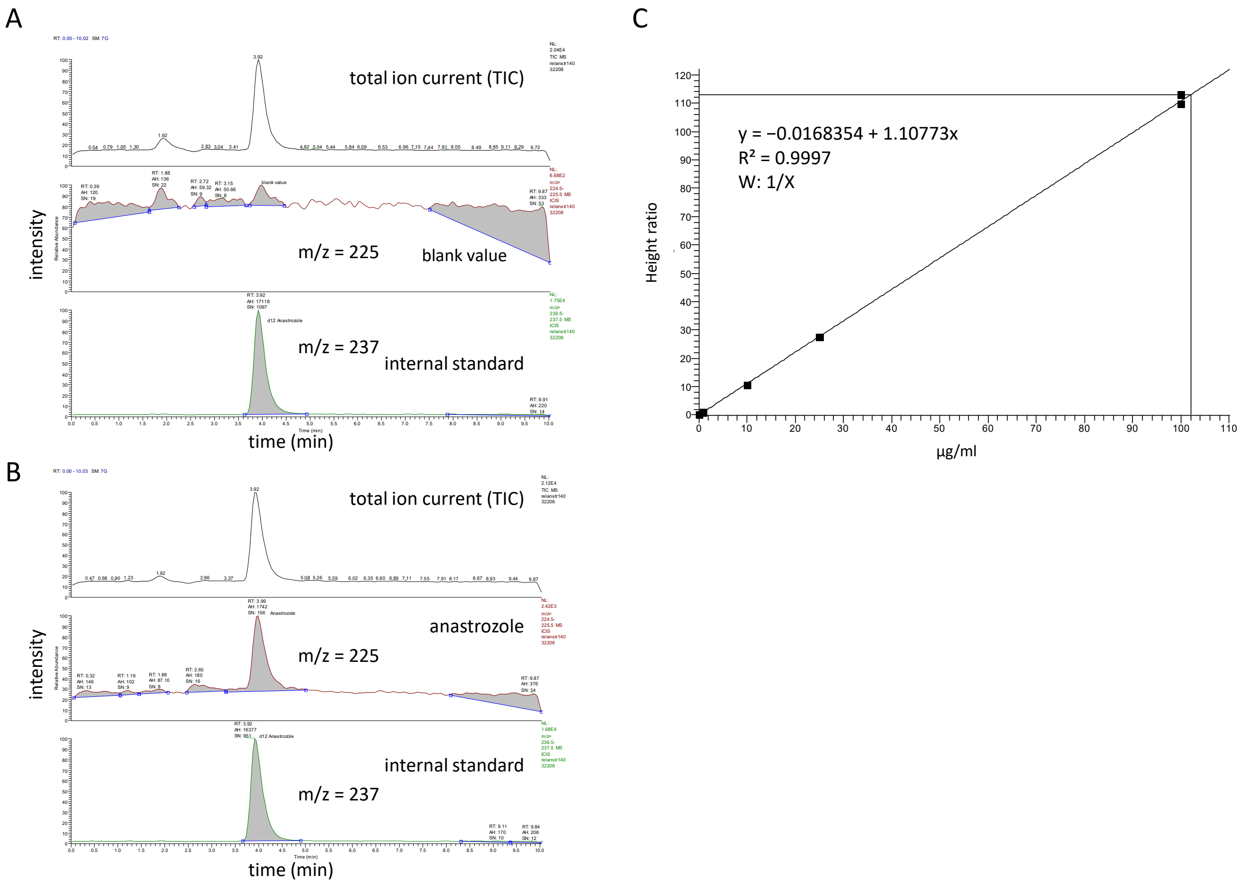

Figure S1. Determination of drug concentrations by liquid chromatography-mass spectrometry (LC-MS). Representative chromatograms of (A) blank value and internal standard, and (B) anastrozole, with the limit of quantitation at 0.1 µg/mL. (C) Calibration curve for anastrozole, in the range of 0.1–100 µg/mL.

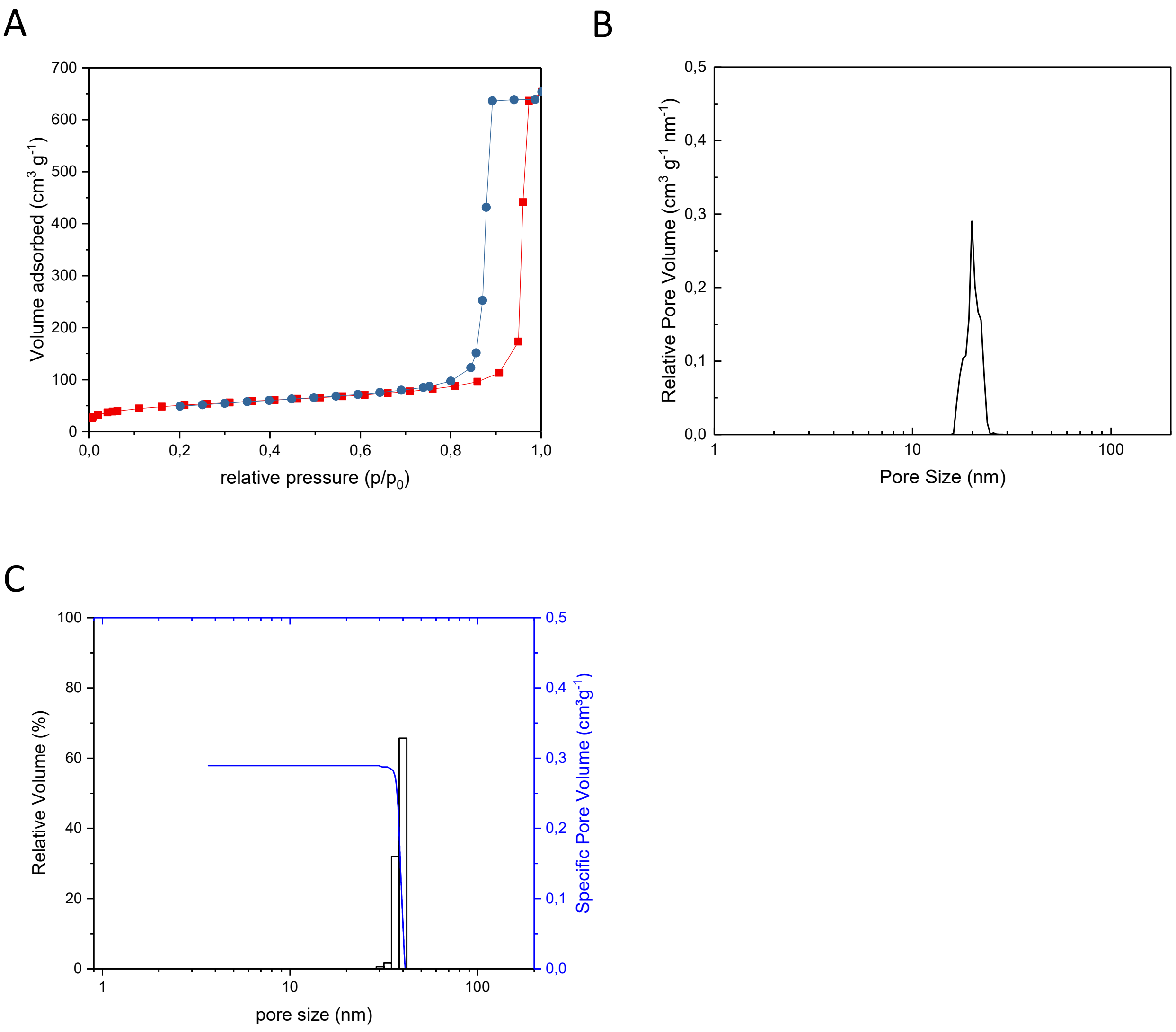

Figure S2. (A) Selected nitrogen adsorption-desorption isotherms and (B) pore size distribution by DFT method (right) of membranes (here: CPG-300-0.87). (C) Selected pore size distribution obtained by mercury intrusion porosimetry of membranes (here: CPG-300-0.40).

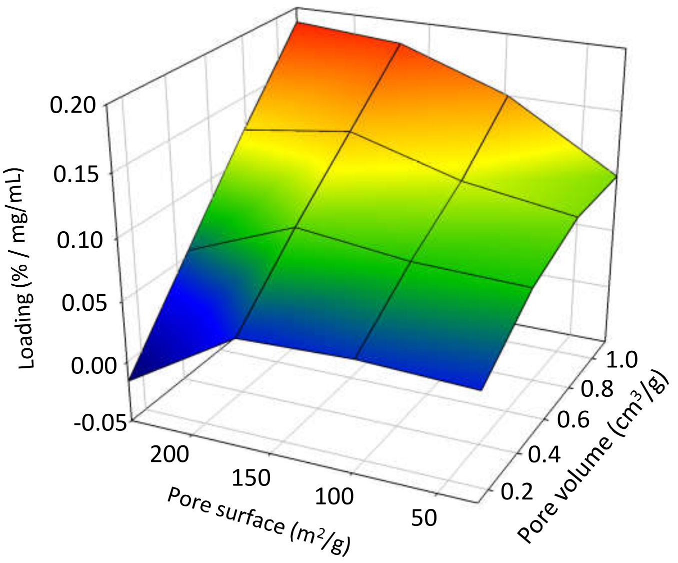

Figure S3. Three-dimensional plot for depicting the dependence of drug loading on pore volume and pore diameter.

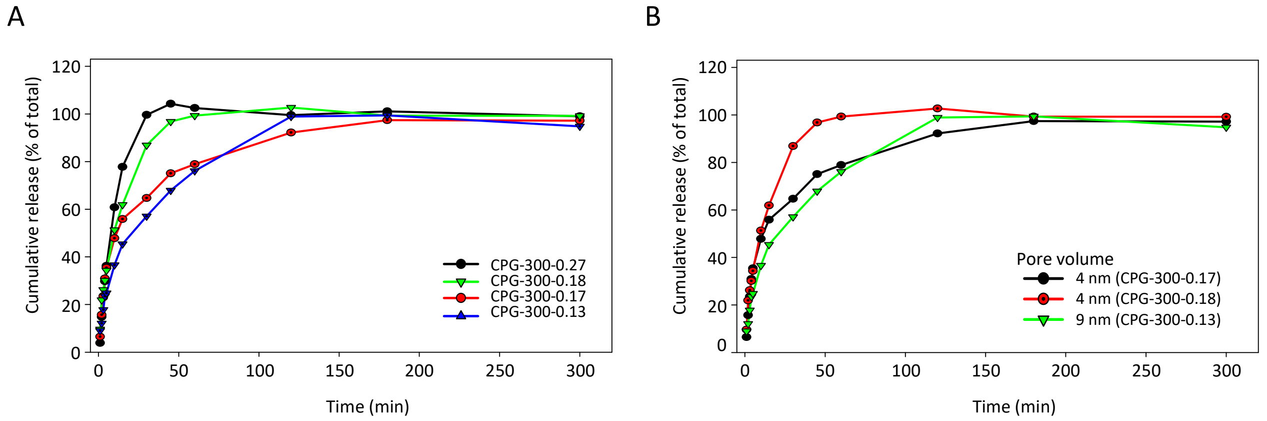

Figure S4. Anastrozole release from sample CPG-300-0.13 with small pore diameter (9 nm) and small pore volume (0.13 cm^3/g).
